# Aggregating Behavior of Phenolic Compounds — A Source of False Bioassay Results?

**DOI:** 10.3390/molecules170910774

**Published:** 2012-09-07

**Authors:** Leena Pohjala, Päivi Tammela

**Affiliations:** 1 Division of Pharmaceutical Biology, Faculty of Pharmacy, University of Helsinki, P.O. Box 56 (Viikinkaari 5 E), FI-00014 Helsinki, Finland; 2 Pharmaceutical Sciences, Department of Biosciences, Åbo Akademi University, BioCity, Tykistökatu 6 A, 20520 Turku, Finland; 3 Centre for Drug Research, Faculty of Pharmacy, University of Helsinki, P.O. Box 56 (Viikinkaari 5 E), FI-00014 Helsinki, Helsinki, Finland; Email: paivi.tammela@helsinki.fi

**Keywords:** flavonoid, bioactivity screening, promiscuous binder, nonspecific inhibition, quercetin, rhamnetin, aggregation, dynamic light scattering

## Abstract

Previous descriptions of quercetin, a widely studied flavonoid, as a frequently reported nonspecific screening hit due to aggregating behavior has raised questions about the reliability of *in vitro* bioactivity reports of phenolic compounds. Here a systematic study on 117 phenolic compounds is presented, concerning their aggregating tendency and the relevance of this phenomenon to obtaining false bioassay results. Fourteen compounds formed aggregates detectable by dynamic light scattering (DLS) when assayed at 10 µM in Tris-HCl pH 7.5. Flavonoids were more prone to aggregation than other phenolic compounds, and the aggregate formation was highly dependent on the vehicle, ionic strength and pH. The compounds were also assayed against three unrelated enzymes in the presence and absence of Triton X-100, and their bioactivity ratios were collected from PubChem database. By comparing these datasets, quercetin and rhamnetin were confirmed as promiscuous inhibitors. In general, flavonoids exhibited also higher bioactivity ratios in the PubChem database than coumarins or organic acids. To conclude, aggregate formation can be controlled with Triton X-100 and this phenomenon needs to be considered when bioassay data is interpreted, but our data indicates that it does not always lead to unspecific inhibition of biological targets.

## Abbreviations

BSA = bovine serum albumin; DLS = dynamic light scattering; DMSO = dimethyl sulfoxide; MOPS = 3-(*N*-morpholino)propanesulfonic acid; PBS = phosphate-buffered saline.

## 1. Introduction

Polyphenolic compounds are a wide class of plant secondary metabolites, produced typically to promote reproduction and for protection against radiation or pathogenic microbes [1]. Flavonoids represent the most common group of polyphenolic compounds, but organic acids and coumarins are also widely distributed in the plant kingdom. Phenolic compounds are present in almost all foods of plant origin, especially onions, apples, berries, tea and red wine [2]. Several epidemiological studies have demonstrated the health benefits of diets rich in phenolic compounds, especially in protection against cardiovascular disease and different cancers [3,4]. The possible mechanisms of the health-promoting action of dietary phenolic compounds have been extensively studied and it has become evident that their pharmacological activities extend far beyond their well-described antioxidative properties. To elucidate the actions of phenolic compounds in more detail, a lot of effort is currently being directed towards assaying individual purified phenolic compounds against isolated target systems considered to be relevant for disease aetiology or pathogenesis. Natural products continue to be an important resource for drug discovery and addressing the challenging features of drug discovery processes from natural products is considered a key factor for better success rates and the maintenance of common interest in the area [5,6,7]. 

Bioassays based on isolated protein preparations typically yield a degree of false positive hits, *i.e.*, compounds whose apparent activity on the target is not reproducible or cannot be confirmed in follow-up assays using other assay formats or methodologies [8]. The proportion of false positives on screening hit lists is highly dependent on the nature of the assay as well as, to some extent, on the chemical collections used as screening material. During recent systematic studies it has been observed that some chemical agents appear in the hit lists more frequently than others. Because these chemicals were noted to inhibit several unrelated protein targets, they have been referred to as promiscuous binders or inhibitors. The general use of such a term stems from the series of articles published by Shoichet and coworkers in early 2000s, in which the concept of aggregation as a common mechanism of nonspecific inhibition by many structurally unrelated frequent hits was introduced [9,10,11]. It was noticed that many such hits share features that are nonclassical in terms of biochemical inhibition of enzymes or receptor proteins, like steep dose-response relationships and the inhibition being highly dependent on experimental parameters (pH, ionic strength and protein concentration). It was demonstrated by dynamic light scattering (DLS) and transmission electron microscopy (TEM) that many of these compounds were not fully solubilised in biochemical buffers but rather formed spherical aggregates of varying diameter. The relationship between aggregate formation and nonspecific inhibition was indicated, among other things, by the elimination of the inhibitory activity by the addition of the non-ionic detergent Triton X-100 to the assay buffer, which also diminished the DLS signals indicating the disintegration of aggregates.

Among the first promiscuous binder reported by McGovern and Shoichet [11] was quercetin, a naturally occurring flavonoid which has been widely investigated for various biological activities. Quercetin was shown to form large aggregates of >1,000 nm in diameter and was nominated as a typical promiscuous inhibitor exhibiting highly condition-sensitive inhibition of various unrelated targets. Since then, flavonoids and polyphenolic compounds in general have been referred to in some literature as aggregating and thus non-leadlike chemical agents [8,12], but no experimental data has been presented in literature to evaluate this issue in detail. The prevalence of aggregate formation among flavonoids and other phenolic compounds is not known, and nor are the potential implications of the aggregating behavior in bioactivity studies. Given the immense interest in flavonoids and other phenolic compounds as therapeutic or protective agents in various pathological conditions the potential for false interpretations of bioassay results needs consideration.

In the current work a systematic study on their aggregating tendency of 117 commercially available pure phenolic compounds and its relevance as a source of biased bioassay results was conducted. Analyses of dynamic light scattering profiles of the compound collection were combined with bioactivity profiling based on database searches and experimental data from enzyme inhibition screens with three unrelated enzymes.

## 2. Results and Discussion

### 2.1. DLS Analysis

#### 2.1.1. Prevalence of Aggregating Behavior among Phenolic Compounds

Dynamic light scattering (DLS) is a widely used method in material sciences to determine particle sizes in solutions. The measurements in general yield two parameters: scattering intensity, which is a representative of particle concentration and average particle size, which is calculated based on the autocorrelation functions of the scattered laser light. Here, DLS was used to detect aggregates in samples in which each of the phenolic compounds was diluted to a final concentration of 10 µM in Tris-HCl buffer (10 mM, pH 7.5). In these measurements, 14 out of the 117 compounds (12%) showed reproducible scattering intensities that were significantly higher than the blank sample. Table 1 lists these 14 compounds and the scattering intensity values and average particle sizes determined in the measurements. 

The average particle sizes for all 14 compounds were >700 nm, except for lauryl gallate and sinapic acid which had average particle diameters of 161 nm and 133 nm, respectively (Table 1). The size range of the other 12 compounds was similar to what has earlier been reported for quercetin, as McGovern *et al*. reported 100 µM quercetin solution to contain aggregates with average diameter >1,000 nm [11]). However, precision of size measurements with DLS is typically compromised with particles of this high size, and no further emphasis was put on analyzing the changes in particle size in this study.

**Table 1:** Scattering intensities and average particle sizes of samples with significant DLS signals in Tris-HCl.

Compound	Intensity (kCounts)	Size (nm)
Acacetin	23.4	3,928
Apigenin	18.9	1,067
Bergapten	16.7	954
Chrysin	53.9	1,710
Coumarin 153	13.5	1,116
Hesperidin	31.7	722
Isorhamnetin	15.9	747
Lauryl gallate	52.3	161
4-Methylumbelliferone	17.1	891
Myricetin	20.7	805
Quercetin	49.5	1,127
Rhamnetin	14.5	1,376
Sinapic acid	33.5	133
Tannic acid	16.3	133
Blank	3.4	N/A

The compounds were diluted into Tris-HCl (pH 7.5) to a final concentration 10 µM and allowed to equilibrate in room temperature for 2 h prior to the measurements. The results represent mean values of three separately prepared samples each measured with 10 DLS readings. Relative standard deviations were 5–10% in all measurements and the values were confirmed as statistically significantly higher than the blank sample by unpaired t-test (*p* < 0.05).

Shoichet and coworkers have also studied the relationship between compound concentration and aggregate formation, and demonstrated the existence of a critical aggregating concentration typical for each aggregating compound [13]. Above this threshold concentration the molar concentration of the monomeric compound remains constant and adding more organic material results in an increased number of aggregates, but does not affect the average aggregate size. Similar characteristics were also observed here, as we confirmed the aggregate formation of some phenolic compounds exhibiting weak DLS signals at 10 µM by running the samples at higher concentrations. For example, 100 µM rhamnetin samples yielded a scattering intensity of 74.3 kCounts/s on average and the average particle diameter of 960 nm, while 100 µM coumarin 153 samples showed a still relatively weak DLS signal, with a mean scattering intensity of 21.6 kCounts/s and average particle diameter 899 nm.

In the current study, DLS analyses were conducted at low micromolar concentration (10 µM) and the results indicate that some phenolic compounds may form DLS-detectable aggregates at this concentration which is typically used in bioactivity studies. Considering the findings on a specific critical aggregating concentration it should be kept in mind that even some of the compounds not reported as DLS-positive here may form aggregates at higher sample concentrations. Furthermore, it has been shown that presence of multiple organic small molecules in the same solution may trigger aggregate formation even when the concentrations of individual compounds in the mixture are below the critical aggregating concentration [14]. Such a concept of total organic load as aggregate promoting factor may have implications also for properties of phenolic compound mixtures such as crude or partially purified extracts of plant material and the process of bioassay-guided fractionation.

The 14 compounds showing significant DLS signals included eight flavonoids (acacetin, apigenin, chrysin, hesperidin, isorhamnetin, myricetin, quercetin and rhamnetin), three coumarins (bergapten, coumarin 153 and 4-methylumbelliferone), two organic acids (sinapic acid and tannic acid) and one gallic acid derivative (lauryl gallate). As the total numbers of flavonoids, coumarins and organic acids was rather close to each other (n = 37 for flavonoids, n = 32 for coumarins and n = 27 for organic acids), these data indicates that aggregation was more frequently observed among flavonoid-structured compounds (eight out of 37, *i.e.*, 22%) than with the other types of phenolic compounds tested (9% for coumarins and 7% of organic acids).

#### 2.1.2. Impact of Experimental Conditions on the Aggregate Formation

As the testing of the 117 compound collection indicated that flavonoids are more prone to aggregate formation than other phenolic compounds tested and previous published data has mentioned quercetin in this respect, we focused on studying in more detail the behavior of a subset of 23 flavonoid-structured compounds and conducted DLS analyses with these compounds under varying conditions, including different buffers, varying ionic strength and pH as well as in the presence of non-ionic detergent. 

Table 2 presents the DLS scattering intensities of 10 µM solutions of these 23 flavonoids diluted in four different aqueous media: Tris-HCl, mQ water, 3-(*N*-morpholino)propanesulfonic acid (MOPS) and phosphate-buffered saline (PBS). As illustrated by the results in the table, the aggregating tendency of flavonoids is highly dependent on the assay buffer. In Tris-HCl, mQ water and MOPS the numbers of DLS-positive samples (showing statistically significantly higher signals than the blank sample) were eight, seven and six, respectively, while only one compound (isorhamnetin) exhibited a significant DLS signal in PBS. 

**Table 2:** DLS Scattering intensities (kCounts) of selected flavonoids in different buffers *^a^*.

Compound	Tris-HCl	mQ water	MOPS	PBS
Acacetin	23.4 *	154.1 *	3.2	4.7
Apigenin	18.9 *	4.2	4.5	5.4
(+)-Catechin	9.2	3.7	5.2	4.3
Chrysin	53.9 *	3.1	4.6	4.8
Daidzein	4.1	7.9	13.1 *	4.4
(−)-Epicatechin	4.8	4.3	4.5	7.1
Flavone	5.3	7.6	11.1 *	3.5
Genistein	9.1	14.8 *	9.8 *	7.4
Gossypin	9.6	4.0	5.2	8.3
Hesperidin	31.7 *	6.7	3.7	5.4
Isorhamnetin	15.9 *	89.3 *	27.1 *	23.3 *
Luteolin	3.5	7.8*	5.7	3.1
Luteolin-7-glycoside	4.5	3.6	4.4	7.0
Morin	4.3	4.7	49.7 *	3.9
Myricetin	20.7 *	31.5 *	9.2	5.1
Naringenin	2.6	3.4	3.2	5.4
Procyanidin B1	8.9	5.7	7.4	3.4
Procyanidin B2	7.9	5.1	3.8	2.3
Quercetin	49.5 *	122.7 *	4.8	4.4
Quercitrin	6.0	2.4	7.5	7.1
Rhamnetin	14.5 *	57.7 *	103.4 *	8.9
Rutin	8.4	2.9	7.2	4.6
Silybin	3.1	2.8	9.7	5.1
Blank	3.4	4.1	2.7	4.7

The compounds were diluted into Tris-HCl, mQ water, MOPS or PBS to a final concentration 10 µM and allowed to equilibrate in room temperature for 2 h prior to the measurements. The results represent mean values of three separately prepared samples each measured with 10 DLS readings. Relative standard deviations were 5–20% and values statistically higher than the blank sample as determined with Student’s t-test are indicated with an asterisk (*).

Furthermore, different flavonoids were identified as DLS-positives in different buffers. For instance, daidzein and morin had detectable DLS signals in MOPS, but yielded scattering intensities similar to a blank sample in three other vehicles. In general, isorhamnetin was the most prominent aggregator among the tested compounds, as it exhibited significant DLS signals in all four vehicles. 

**Figure 1:**
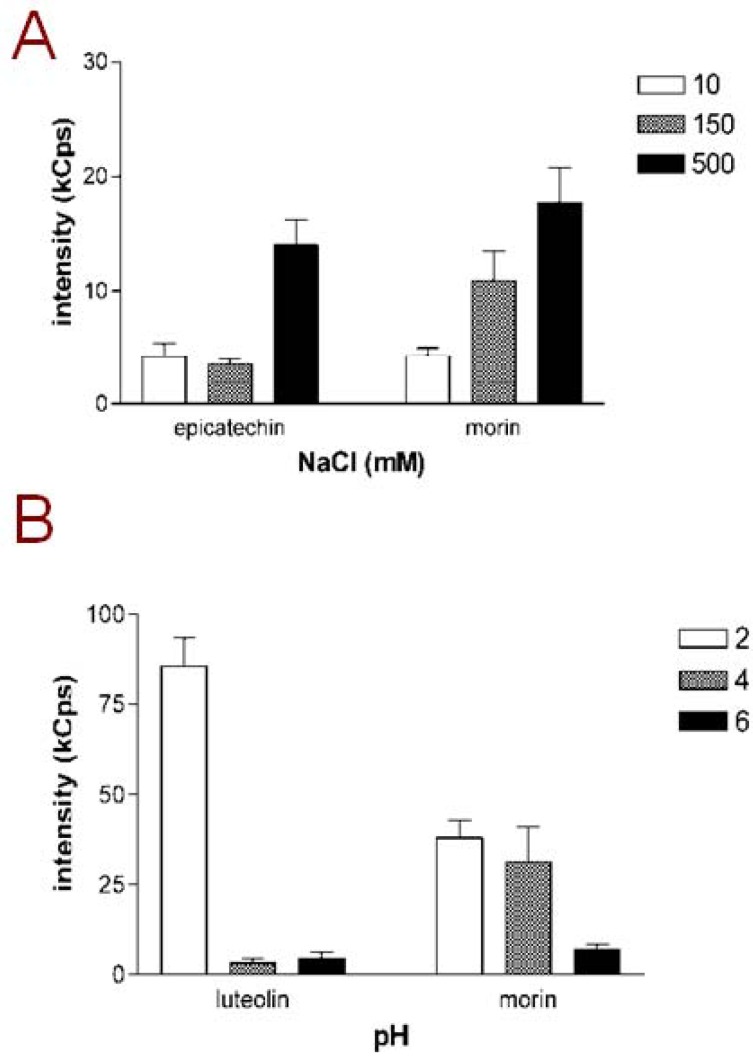
Aggregating behavior of flavonoids is dependent on buffer conditions. (**A**) Scattering intensities of epicatechin and morin, selected as representative examples, in different NaCl concentrations; (**B**) Scattering intensities of luteolin and morin, selected as representative examples, at different pH adjusted with maleic acid. Compound concentration in all measurements was 10 µM and mQ water was used as the vehicle for sample dilutions. Results represent mean and SD of three separately prepared samples each measured with 10 DLS readings.

Next, the effect of buffer ionic strength on aggregating tendency was investigated by preparing 10 µM flavonoid samples in mQ water with 10 mM, 150 mM or 500 mM NaCl. Out of the 23 flavonoids tested, six were DLS-positive in mQ water (acacetin, genistein, isorhamnetin, myricetin, quercetin and rhamnetin; Table 2). In addition to these six flavonoids, daidzein and morin gave DLS signals in 150 mM NaCL, while a total of 12 compounds were DLS-positive in the presence of 500 mM NaCl. In Figure 1A, the scattering intensities of samples with epicatechin and morin, selected as representative examples, are presented. Both of these compounds exhibited increasing DLS signals in the higher NaCl concentration. However in some cases the scattering intensity did not show linear increase with increasing NaCL concentrations: acacetin, for example, had a scattering intensity of 154.1 kCounts/s in mQ water and 210.6 kCounts/s in 150 mM NaCl but only 47.5 kCounts/s in 500 mM NaCl. Such a nonlinear correlation between ionic strength and the scattering intensity may relate to findings from studies on other aggregating small molecules which have indicated that changing the vehicle ionic strength may affect not only the particle concentration but also the particle size [9,10]. In other words, increased ionic strength may cause a redistribution of the organic material into larger aggregates which are fewer in numbers than those formed in the solution with lower ionic strength. However, given the exceptionally large size of flavonoid aggregates compared to many other aggregate-forming small molecules we could not reliably detect changes in particle size in this study. 

The effect of pH on the aggregating tendency of flavonoids was investigated by preparing 10 µM samples of the compounds into maleic acid buffers adjusted to different pH. DLS analysis of these samples showed that also pH has a major effect on the aggregating tendency of most of the flavonoids. Figure 1B shows the scattering intensities of luteolin and morin, selected as representative examples, in maleic acid buffers at pH 2, 4 and 6. As illustrated by the columns, both luteolin and morin form a high number of aggregates at pH 2, while the aggregate formation is less intense, or in the case of luteolin, not detectable at higher pH. This pattern was shared by all flavonoids listed in Table 2, except for genistein, which did not yield DLS signal in any of the pH tested. The dependence of aggregate formation has also been indicated with one earlier study on hydrophobic small molecules [15]. The analysis of nonnucleoside reverse transcriptase inhibitors (NNRTIs) designed as anti-HIV agents demonstrated that aggregate numbers and sizes of these compounds are significantly changed depending on vehicle pH, and by correlating the aggregation data in solutions mimicking gastrointestinal fluids with the known *in vivo* bioavailability properties of these drug molecules the authors suggested that the aggregation profiles of these compounds explain the differences in their oral bioavailabilities. Also dietary flavonoids are known to have poor oral bioavailabilities owing to their intense first-pass metabolism but also to low water-solubility [16] and this role of the environment-dependent aggregation as one contributing factor can be speculated.

**Figure 2:**
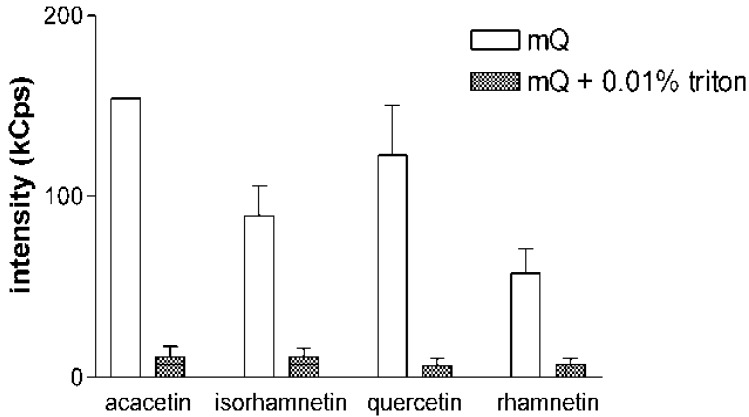
Addition of non-ionic detergent eliminates the DLS signal of flavonoids. Scattering intensities of acacetin, isorhamnetin, quercetin and rhamnetin, selected as representative examples, in pure mQ water and in mQ water with 0.01% Triton X-100. Sample concentration was 10 µM in all measurements. Results represent mean and SD of three separately prepared samples each measured with 10 DLS readings.

One of the hallmarks of small molecule aggregates and promiscuous inhibition is the sensitivity to detergents, and supplementation of assay buffers with low detergent concentrations is suggested to control aggregate-borne false positive results in several bioassay protocols [17,18]. To confirm the nature of the DLS signals observed in the current study, the 10 µM flavonoid samples were run in DLS analysis in the presence or absence of 0.01% Triton X-100, a nonionic detergent that has previously been reported to efficiently solubilize small molecule aggregates [9,10]. As shown in Figure 2 for acacetin, isorhamnetin, quercetin and rhamnetin, the addition of Triton X-100 decreases the DLS signals of the samples into the level of a blank sample. This phenomenon was consistent in all experiments and with all the compounds tested in the study. These experiments also confirmed the reversible nature of the aggregate formation by flavonoids, as similar results were obtained when the compounds were first diluted in mQ water and allowed to equilibrate for 2 h before Triton X-100 was added into the solution. 

### 2.2. Screening for Enzyme Inhibition

Based on our DLS data, it was evident that aggregate formation occurs in solutions of flavonoids and some other phenolic compounds when they are diluted in buffers typical of experimental *in vitro* studies. The implications of such behavior on bioassay results were examined by using enzyme activity assays for three unrelated enzymes: lactate dehydrogenase (LDH; EC 1.1.1.27), α-chymotrypsin (EC 3.4.21.2) and β-lactamase (EC 3.5.2.6). The three enzymes were obtained from commercial sources and their enzymatic activities were assayed using published and widely applied protocols (see Experimental section for details). The 117 phenolic compounds in our collection were assayed against these three enzymes both in the absence and in the presence of 0.01% (v/v) Triton X-100 which is known to solubilize small molecule aggregates (Figure 2) [9,10,11,17,18]. This detergent concentration was also confirmed not to affect the activity of the studied enzymes (data not shown).

**Figure 3:**
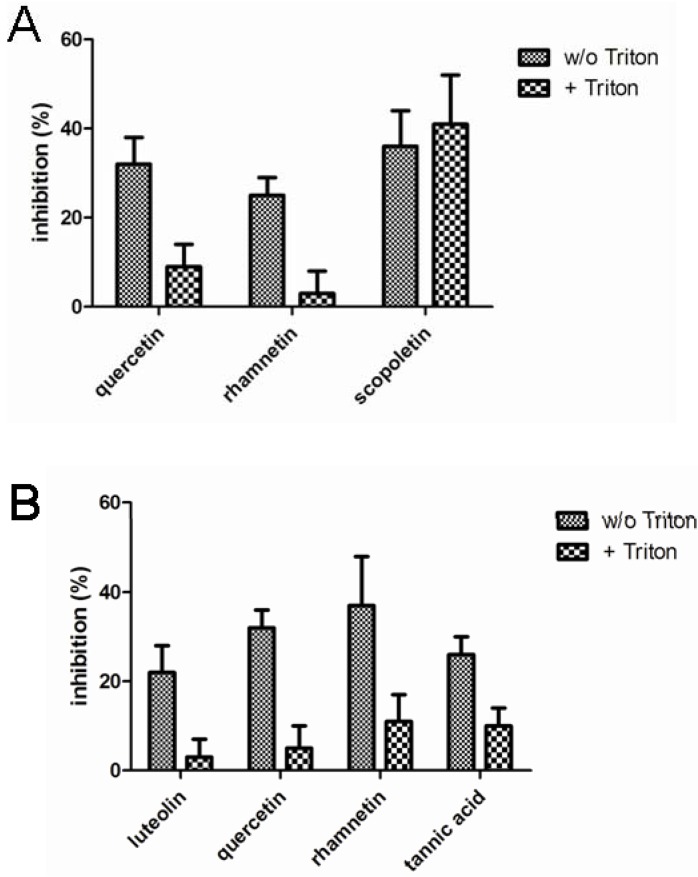
Inhibition of lactate dehydrogenase (**A**) and chymotrypsin (**B**) enzymatic activity by the phenolic compounds (10 µM) identified as hits in the absence of Triton X-100. The results represent mean and standard deviation values of six replicates both in the absence and in the presence of 0.01% Triton X-100.

Figure 3 presents the compound identified as confirmed hits in the enzymatic assays. In LDH inhibition assays, quercetin, rhamnetin and scopoletin inhibited the enzymatic activity in the absence of Triton X-100 while only scopoletin maintained its inhibitory activity when the screen was performed in the presence of Triton X-100. In the chymotrypsin assays, on the other hand, luteolin, quercetin, rhamnetin and tannic acid were indentified as confirmed screening hits in the absence of Triton X-100 but neither any of them nor any other of the studied compounds, showed inhibitory activity when the assay buffer was supplemented with Triton X-100. None of the 117 compounds studied inhibited β-lactamase in our assay either in the presence or absence of Triton X-100.

In the recent study where quercetin was reported as a promiscuous inhibitor, it inhibited β-lactamase with an IC_50_ value of 4 μM, chymotrypsin with an IC_50_ value of approximately 100 µM and a third enzyme, malate dehydrogenase, with an IC_50_ value of 6 µM [11]. While the inhibition of chymotrypsin in our study by quercetin seemed slightly more efficient than reported earlier (approximately 30% inhibition at 10 µM), we were not able to detect inhibition (neither detergent-sensitive nor insensitive) of β-lactamase by quercetin in low micromolar concentrations. As pointed out in earlier studies on promiscuous inhibition, this phenomenon is highly dependent on assay conditions, and differences in assay buffer composition and total protein content may explain the nonreproducible results between our study and the earlier reports in this respect. McGovern and Shoichet [11] also reported that quercetin’s IC_50_ value against β-lactamase increased over 50-fold when tested against a 10 times higher enzyme concentration and addition of bovine serum albumin into the assay buffer had a similar effect. Thus, differences in the total protein concentration due to different specific activities of enzyme preparations may affect the assay sensitivity to promiscuous-type inhibition. Within the introduction of the concept, possible mechanisms for the promiscuous enzyme inhibition of aggregates were studied, including formation of covalent adducts and local denaturation of the catalytic sites [9,10]. However, most experimental data supported the idea that aggregates act as nonspecific inhibitors by sequestering enzyme molecules by absorbing or adsorbing them within their structure. This hypothesis was also supported by the findings that increasing protein concentration in the assay significantly weakens the potency values of promiscuous inhibitors even though the enzyme concentration remained several orders of magnitude below the small molecule monomer concentration.

To classify a small molecule as a promiscuous inhibitor, the criteria used in the literature include DLS signal and detergent sensitive inhibition of at least two out of three unrelated enzymes tested. Applying such criteria in the current dataset, quercetin and rhamnetin were the only compounds that met this definition. Interestingly, these two flavonoids are closely related and differ from each other solely by one methyl group: in rhamnetin the hydroxyl group in position C7 is methylated, while all the hydroxyl groups in quercetin are free. However, these experiments revealed several phenolic compounds that formed DLS detectable aggregates but did not behave as promiscuous enzyme inhibitors. 

In previously reported work introducing the concept of aggregate formation as a common mechanism of frequent hits or promiscuous binders, large chemical collections were assayed for enzymatic activities and the hits were examined for aggregating behavior using DLS and other methodologies [9,10,11,19]. In these studies, a high proportion of the hits were found to be DLS-detectable, leading to the conclusion that aggregate formation is a typical feature leading to false positives in bioactivity screens. In the current study aggregate formation was examined systematically for all compounds included in the study and sample selection for DLS was thus independent of the biological activities observed. Although a limited number of compounds were studied, our data demonstrates that aggregating behavior can be observed with phenolic compounds not showing features of promiscuous inhibitor. Such a phenomenon was not visible from the earlier studies since DLS analyses were only carried out on the screening hits. Consistent with earlier studies, the cases of quercetin and rhamnetin illustrate that aggregation may lead to promiscuous inhibition profiles, but the data presented here also indicate that aggregate formation does not automatically manifest itself as false positive bioactivity results. In fact, a question may be raised whether aggregate formation may alternatively result in false negative results due to the lower concentration of the compound available in the solution. This possibility remains fully speculative but might have implications when considering for instance the interpretation of structure-activity relationship analyses of phenolic compounds against different pharmacological targets. 

### 2.3. Bioactivity Profiling Based on the PubChem Database

As nonspecific inhibition due to aggregate formation is sensitive to experimental conditions, the generalization of data from individual experimental work such as the current study is difficult. On the other hand, examining the bioactivity profiles based on larger datasets such as those available in public databases may reveal more general patterns of nonspecific inhibition. To this end, a database search was conducted on the bioactivity data reported for the compounds in the PubChem BioAssays database [20]. This is a publicly available resource currently containing data from more than 500,000 bioactivity screening campaigns, with a total of approximately 740 million data points [21]. The database was searched for each of the 117 compounds included in our collection and bioactivity summary data was collected. Bioactivity ratios for each compound were determined by dividing the number of records in which the compound was reported as active by the total number of bioassay records in which the compound had been included. Altogether 112 of the 117 compounds could be found in the database and ten compounds with a total number of bioassay records less than 10 were excluded from the analysis. By using this exclusion criterion, a total of 102 compounds were used for the analyses. 

Table S1 (Supplementary Information) lists the database search results and activity ratios for all the compounds in this study, and the compounds with the highest activity ratios are presented in Table 3. The mean value calculated as the average of all determined activity ratios was 0.1097 and the median value was 0.0772. While most of the compounds included in the study had an activity ratio of 0.1, some compounds were showing clearly higher values, up to 0.76 by quercetagetin. 

**Table 3:** Top 10 phenolic compounds ranked according to the activity ratio extracted from the PubChem Bioassays database.

Compound	Active(total) ^a^	Activity ratio
Quercetagetin	16 (21)	0.76
3,5-Dihydroxybenzoic acid	10 (15)	0.67
Cyanidin chloride	5 (11)	0.45
(–)-Epicatechin gallate	42 (117)	0.36
Quercetin	419 (1,290)	0.32
(–)-Epigallocatechin gallate	160 (576)	0.28
Resveratrol	410 (1,687)	0.25
Tannic acid	30 (127)	0.24
α-Naphtoflavone	31 (135)	0.23
Myricetin	171 (782)	0.22

^a ^Numbers of entries in the PubChem BioAssays database in July 2012 in which the compound was reported as active and the total number of bioassay entries in which the compound was included.

Among the ten phenolic compounds with the highest bioactivity ratios seven compounds are flavonoids, including also quercetin and quercetagetin (6-hydroxyquercetin). An interesting feature was also that both (–)-epicatechin gallate and (–)-epigallocatechin gallate are within the top ten compounds (bioactivity ratios 0.36 and 0.28, respectively), while the corresponding compounds (–)-epicatechin and (–)-epigallocatechin have bioactivity ratios of 0.07 and 0.11, respectively (Supplementary Table S1). Tannic acid and resveratrol were also among the compounds with highest bioactivity ratios but none of the 32 coumarins included in the study had such high bioactivity ratios. 

When the bioactivity ratios of the 102 compounds were analyzed by phytochemical class, a significant difference was observed between the groups. While flavonoids (n = 33) had an average bioactivity ratio of 0.14, the value for coumarins (n = 26) was 0.06. Mean values for organic acids and the subset classified as other phenolic compounds were closer to the general average of the studied compound collection (activity ratio 0.12 for organic acid and 0.09 for other phenolic compounds, n = 24 and 19, respectively). 

Comparing the database search results to our experimental data provided further support for the hypothesis of quercetin being a aggregate-based nonspecific inhibitor of several unrelated targets. Quercetin has been reported as active in 419 of the 1,290 bioassays in which it had been tested by July 2012, thus yielding a ratio of 0.32. On the other hand, rhamnetin has been reported as active in 18 out of 175 bioassays, which gives an activity ratio of 0.10 which is close to the overall average of the 102 compounds included in the analysis. The average bioactivity ratio of the 14 phenolic compounds that we originally detected as aggregators at 10 µM (Table 1) is 0.125 which is slightly higher than the general average but this difference is not strikingly large. 

The database bioassay records represent a wide spectrum of different assay protocols from simple biochemical enzyme inhibition assays to more complex cell-based setups and thus also the nonspecific hits may result from different sources. Cytotoxic properties of some small molecules represent one such a source in primary screens with cell-based assays. For instance, a synthetic coumarin derivative 7-diethylamino-3-thenoylcoumarin has-been reported as active in 13 PubChem bioassay records, 12 of which were cell-based primary screens on different targets. Based on our own screening data on cell-based assays we have observed that this synthetic coumarin significantly decreases mammalian cell viability at low micromolar concentration [22] and thus the relatively high bioactivity ratio (0.16) compared to coumarin average may result from nonspecific toxicity on the assayed cell line. Furthermore, assay artifacts may rise from more specific interactions of small molecules with assay components. For instance, resveratrol, a widely investigated stilbene abundant in red wine, has been reported to inhibit firefly luciferase in low micromolar concentration and is therefore prone for being identified as an apparent hit in reporter gene assays utilising this enzyme [23]. As illustrated by these examples, it is worth noticing that aggregate formation is not the only source for potential false interpretations of bioassay data and drawing conclusions on to which extent aggregating behavior leads to false bioassay results is not straightforward based on the database searches. 

In summary the results presented in the current work demonstrate that flavonoids are more prone to aggregate formation than the set of coumarins studied and that flavonoids also exhibit higher bioactivity ratios in PubChem database than coumarins. The group of organic acids appeared between flavonoids and coumarins in both respects, showing some signs of aggregating behavior and the average bioactivity ratio close to the general average of the 102 studied compounds. Virtually all flavonoids studied formed DLS-detectable aggregates in some of the experimental conditions tested. In the case of quercetin and rhamnetin, the aggregating behavior was also shown to result in promiscuous inhibition characteristics. 

Studies on high-throughput screening campaigns have indicated that aggregate-forming nonspecific inhibitors may represent up to 95% of screening hits [17,19]. Given the high proportion of these unfavorable hit candidates, the removal of aggregating compounds from screening collections has been suggested [24,25]. However, such an action is not encouraged by the finding that also some known drugs form DLS-detectable aggregates and act as promiscuous inhibitors when they are used at high concentrations in biochemical assays [26]. In fact, the proportion of aggregating compounds among the studied known drugs was approximately 8%, which is in the same range as the prevalence of aggregating behavior observed in the current study for phenolic compounds (approximately 12%). As pointed out by the authors of earlier aggregation studies, from a physicochemical point of view it is not surprising that some relatively hydrophobic small molecules form aggregates when they are put in aqueous media with no protein and membrane components and/or with nonphysiological pH or ionic strength. However, aggregating behavior warrants consideration when designing bioassays and interpreting data generated by them. The detergent test is a widely accepted means to control aggregate-borne artifacts and when applicable, its use can be recommended for *in vitro* studies on phenolic compounds. However, not all bioassay protocols are compatible with detergent supplementation, and addition of bovine serum albumin (BSA) in the assay buffer has been suggested as an alternative for detergents [9]. We have earlier observed that the presence of β-lactoglobulin from bovine milk, which has a binding pocket for hydrophobic small molecules, is able to diminish the DLS signals generated by aqueous solutions of synthetic retinoid analogues [27]. Within the current work on aggregating behavior of phenolic compounds, attempts were made for DLS measurements in the presence of BSA or by using fetal bovine serum (FBS) as a vehicle were made but due to unstable background values no reliable data was obtained. Thus, we could not obtain experimental support for the aggregate-resolving effects of protein addition but the putative mechanism of nonspecific protein sequestering by aggregates and the experimental findings on the decreased assay sensitivity to promiscuous inhibitors upon BSA addition indicate that it may provide a good alternative for detergents in this respect.

## 3. Experimental

### 3.1. Chemicals

The complete list of an in-house collection of 117 phenolic compounds used in the study is presented in the Supplementary Information (Table S1). The collection contained 37 flavonoids, 32 coumarins, 27 organic acids and 21 other phenolic compounds. All compounds in the collection were purchased from commercial sources (see Supplementary Information for details) and dissolved in dimethyl sulfoxide (DMSO) at a concentration of 20 mM. The stock solutions were stored at −20 °C and none of the aliquots had been subjected to more than 10 freeze-thaw cycles. 

### 3.2. Dynamic Light Scattering (DLS)

All phenolic compounds included in the study were diluted in 10 mM Tris-HCl, pH 7.5 to yield a final concentration of 10 µM. For the experiments with flavonoids in different buffers, each of the compounds tested was diluted in either10 mM Tris-HCl (pH 7.5), mQ water (pH 7.0), 10 mM MOPS (pH 7.0) or PBS (with Ca^2+^ and Mg^2+^, pH 7.2). In the experiments with different pH, 10 mM maleic acid buffer was prepared in mQ water and the pH of the buffer aliquots was adjusted with HCl and NaOH to 2, 4 or 6. In all experiments, the samples were allowed to stand at room temperature for 2 h before the DLS measurements. The measurements were conducted with Zetasizer 300SH instrument (Malvern Instruments Ltd.; Worcestershire, UK). Laser wavelength 633 nm was used for all DLS measurements as none of the samples absorbed light at this wavelength (measured with Varioskan Flash plate reader, Thermo Fisher Scientific, Waltham, MA, USA). Three replicates were prepared from all samples, each measured with 10 repeated runs. 

### 3.3. Enzyme Assays

All enzyme assays were conducted in 10 mM Tris-HCl, pH 7.5, both in the presence and absence of 0.01% Triton X-100. In all experiments, the aliquots of substrate solutions were first mixed with the phenolic compounds (final concentration 10 µM) and the reaction was initiated upon addition of enzyme in these aliquots within 10 min of compound addition. When Triton X-100 was used, it was added into the substrate solutions prior to the addition of phenolic compounds or enzymes. The experiments with and without Triton X-100 were conducted pairwise in the same day and using the same aliquots of compound stock solutions.

### 3.4. Lactate Dehydrogenase Assay

LDH activity was assayed using a protocol modified from [28] based on a substrate solution consisting of 54 mM (+)-lactic acid, 1.3 mM NAD^+^, 0.66 mM iodonitrotetrazolium chloride (INT) and 0.28 mM phenazin methosulfate, all purchased from Sigma (St. Louis, MO, USA) and dissolved into the assay buffer. Lactate dehydrogenase from porcine heart (Sigma) was dissolved into the assay buffer and the reaction was initiated by mixing equal amounts of substrate solution to yield a final enzyme concentration of 1 U/mL. After 30 min incubation in RT with continuous shaking, the enzymatic reaction was stopped with 0.1 M acetic acid and the absorbance was read at 490 nm (Victor plate reader, PerkinElmer Life and Analytical Sciences, Turku, Finland).

### 3.5. α-Chymotrypsin Assay

The enzymatic activity was assayed as described in McGovern *et al.* [9] with slight modifications. Succinyl-Ala-Ala-Pro-Phe-*p*-nitroanilide (Sigma) used as the substrate was prepared as 20 mM stock solution in DMSO and diluted to a final concentration of 200 µM into the assay buffer. Chymotrypsin from bovine pancreas (Sigma) was dissolved into the assay buffer and used at a final concentration of 1.5 U/mL. Formation of the colored cleavage product was monitored at 410 nm with the Varioskan Flash.

### 3.6. β-lactamase Assay

The β-lactamase substrate nitrocefin (Calbiochem, San Diego, CA, , USA) was prepared as a 20 mM stock solution in DMSO and diluted to a final assay concentration 150 µM in the assay buffer. β-lactamase enzyme from *Pseudomonas aeruginosa* (Sigma) was dissolved in the assay buffer. After 60 min enzymatic reaction, the hydrolysis product of nitrocefin was detected with absorbance readout at 482 nm (Varioskan Flash). 

### 3.7. PubChem Database Analysis

Bioassay data available in the PubChem BioAssays database was collected in July 2012 for all compounds included in the study by searching the compounds by name and CAS number in the database. The total number of bioassay entries in which the compound was included, as well as the number of bioassays entries in which the compound was reported to be active were collected. Compounds for which less than 10 bioassay entries had been reported were not included in any further analyses. The bioactivity ratio for each compound was determined as the ratio of the bioassays in which the compound was reported as active and the number of all reported bioassays for the compound.

### 3.8. Statistical Analyses

All samples reported as DLS positive or as enzyme inhibitors were confirmed to have result values with statistically significant difference to the corresponding blank samples by Student’s t-test performed with GraphPad Prism 5.0 software, *p* < 0.05 in each case.

## 4. Conclusions

According to our data, flavonoids are more prone to aggregating behavior in biochemical assay conditions than other phenolic compounds tested, and the studied flavonoids also exhibit a significantly higher bioactivity ratio in the PubChem BioAssays database data than the other phenolic compounds did. However, the comparison of the aggregate formation with published bioactivity profiles and our own data from enzyme assays suggests that this feature does not necessarily manifest itself as false positive bioassay results. On the other hand, use of small amounts of nonionic detergent such as Triton X-100 is a simple means for eliminating the potential bias arising from aggregating tendency, and when applicable, it can be used for additional confirmation of bioassay results obtained with phenolic compounds. The need for careful design of *in vitro* bioassays and the use of specific control experiments and tests is emphasized by these findings. 

## Supplementary Materials

Supplementary materials can be accessed at: http://www.mdpi.com/1420-3049/17/9/10774/s1.
