# Supplementary material for: Aggregating Behavior of Phenolic Compounds — A Source of False Bioassay Results?
Source: Molecules. 2012 Sep 7;17(9):10774–90. doi: 10.3390/molecules170910774 (PMC6268869; doi:10.3390/molecules170910774)
Supplement: Supplementary file 1 [file molecules-17-10774-s001.pdf]

Supplementary Information

Aggregating behavior of Phenolic Compounds: A Source of False Bioassay Results?

Table S1. List of the phenolic compounds used in the study and their bioactivity summary data in PubChem BioAssays database. (PubChem search).

Chemical name	MW	Supplier	active	total	ratio (actuve/total)
Acacetin	284.3	Carl Roth GmbH, Germany	41	520	**0.079**
Alphanaphthoflavone	272.9	Acros, USA	31	135	**0.230**
Apigenin	270.2	Fluka, Switzerland	146	1062	**0.137**
L-ascorbic acid	176.1	Sigma, Germany	67	780	**0.086**
Benzoic acid	122.1	Merck, Germany	24	429	**0.056**
3-Benzoylbenzo(F)coumarin	300.3	Acros, USA	N/A	N/A	
3-(2-Benzoxazolyl)umbelliferone	279.2	Fluka, Switzerland	8	73	**0.110**
Bergapten	216.2	Carl Roth GmbH, Germany	21	590	**0.036**
Butylated hydroxyanisole	180.2	Sigma, Germany	N/A	N/A	
Caffeic acid	180.2	Sigma, USA	101	1101	**0.092**
(+)-Catechin	290.3	Sigma, USA	9	305	**0.030**
Catechol	110.1	Sigma, Germany	70	639	**0.110**
Chrysin	254.3	Extrasynthese, France	84	791	**0.106**
o-Coumaric acid	164.2	Sigma, USA	6	35	**0.171**
Coumarin 102	255.3	Acros, USA	0	2	**0.000**
Coumarin 30	347.4	ICN, USA	127	761	**0.167**
Coumarin 7	333.4	Acros, USA	158	796	**0.198**
Coumarin	146.2	Merck, Germany	38	916	**0.041**
Cyanidin chloride	322.7	Carl Roth GmbH, Germany	5	11	**0.455**
Daidzein	254.3	Extrasynthese, France	61	785	**0.078**
Daphnetin	178.2	Extrasynthese, France	27	240	**0.113**
7-Diethylamino-3-thenoylcoumarin	327.4	Acros, USA	13	82	**0.159**
2,3-Dihydroxybenzoic acid	154.1	Fluka, Switzerland	5	327	**0.015**
2,5-Dihydroxybenzoic acid	154.1	Fluka, Switzerland	5	223	**0.022**
2,6-Dihydroxybenzoic acid	154.1	Fluka, Switzerland	2	14	**0.143**
3,4-Dihydroxybenzoic acid	154.1	Fluka, Switzerland	34	574	**0.059**
3,5-Dihydroxybenzoic acid	154.1	Fluka, Switzerland	10	15	**0.667**
2,5-Dimethylphenol	122.2	Fluka, Switzerland	1	141	**0.007**
Ellagic acid	302.2	Sigma,USA	171	865	**0.198**
(−)-Epicatechin	290.3	Sigma,USA	44	658	**0.067**
(−)-Epicatechin gallate	442.4	Extrasynthese, France	42	117	**0.359**
(-)-Epigallocatechin	306.3	Extrasynthese, France	18	158	**0.114**
(−)-Epigallocatechin gallate	458.4	Extrasynthese, France	160	576	**0.278**
Ethoxyquin	217.3	Sigma, USA	22	543	**0.041**
Ferulic acid	194.2	Extrasynthese, France	30	501	**0.060**

Table S1. *Cont.*

Chemical name	MW	Supplier	active	total	ratio (actuve/total)
Flavone	222.3	Carl Roth GmbH, Germany	46	596	**0.077**
Gallic acid	170.1	Sigma, USA	64	361	**0.177**
Genistein	270.2	Extrasynthese, France	214	1258	**0.170**
Gitoxigenin	390.5	Carl Roth GmbH, Germany	22	81	**0.272**
Gossypin	648.5	Extrasynthese, France	1	3	**0.333**
Hamamelitannin	484.4	Extrasynthese, France	2	13	**0.154**
Hesperidin	610.6	Extrasynthese, France	8	442	**0.018**
2-Hydroxyacetophenone	136.2	Fluka, Switzerland	0	8	**0.000**
3-Hydroxyacetophenone	136.2	Fluka, Switzerland	1	10	**0.100**
4-Hydroxyacetophenone	136.2	Fluka, Switzerland	3	19	**0.158**
2-Hydroxyphenylacetic acid	152.2	Fluka, Switzerland	1	8	**0.125**
3-hydroxyphenylacetic acid	152.2	Fluka, Switzerland	1	9	**0.111**
4-Hydroxyphenylacetic acid	152.2	Fluka, Switzerland	14	328	**0.043**
Hydroquinone	110.1	Fluka, Switzerland	96	531	**0.181**
Isopropyl gallate	212.2	Lancaster, UK	2	11	**0.182**
2,4-Dihydroxylbenzoic acid	154.1	Fluka, Switzerland	9	435	**0.021**
Isorhamnetin	316.3	Extrasynthese, France	15	80	**0.188**
Kaempferol	286.3	Extrasynthese, France	106	789	**0.134**
Khellin	260.2	Carl Roth GmbH, Germany	15	809	**0.019**
Lauryl gallate	338.5	Fluka, Switzerland	9	73	**0.123**
Leucocyanidin	306.3	Carl Roth GmbH, Germany	0	7	**0.000**
Luteolin	286.3	Extrasynthese, France	164	842	**0.195**
Luteolin-7-glucoside	448.4	Extrasynthese, France	33	333	**0.099**
Malvin chloride	691	Extrasynthese, France	0	66	**0.000**
Methyl gallate	184.2	Fluka, Switzerland	17	577	**0.029**
2'-Methoxy-a-naphthoflavone	302.3	ICC, USA	N/A	N/A	
Morin dihydrate	338.3	Carl Roth GmbH, Germany	74	706	**0.105**
Myricetin	318.3	Extrasynthese, France	171	782	**0.219**
Naringenin	272.3	Sigma, Germany	40	830	**0.048**
4-Methyl pyrocatechol	124.1	Merck, Germany	5	152	**0.033**
Nordihydroguaiaretic acid	302.4	Fluka, Switzerland	139	742	**0.187**
Octyl gallate	282.3	Fluka, Switzerland	10	77	**0.130**
Phthalic acid	166.1	Merck, Germany	1	415	**0.002**
Procyanidin B1	578.5	Extrasynthese, France	1	15	**0.067**
Procyanidin B2	578.5	Extrasynthese, France	4	48	**0.083**
n-Propyl gallate	212.2	Sigma, USA	11	164	**0.067**
Protocatechuic acid	154.1	Carl Roth GmbH, Germany	34	574	**0.059**
Pyrogallol	126.1	Riedel-de Haën, Germany	42	315	**0.133**
Quercetagetin	318.2	Carl Roth GmbH, Germany	16	21	**0.762**
Quercetin	338.3	Merck, Germany	419	1290	**0.325**

Table S1. *Cont.*

Chemical name	MW	Supplier	active	total	ratio (actuve/total)
Quercetin-3,7,3',4'-tetramethylether	358.4	Extrasynthese, France	6	94	**0.064**
Quercitrin dihydrate	484.4	Carl Roth GmbH, Germany	13	130	**0.100**
Rosmarinic acid	360.3	Extrasynthese, France	57	351	**0.162**
Rutin	664.6	Merck, Germany	41	506	**0.081**
D(−)-Salicin	286.3	Carl Roth GmbH, Germany	6	497	**0.012**
Salicylic acid	138.1	AnalaR, UK	51	769	**0.066**
Silybin	482.4	Carl Roth GmbH, Germany	15	548	**0.027**
Sinapic acid	224.2	Fluka, Switzerland	6	326	**0.018**
Sinigrin monohydrate	415.5	Carl Roth GmbH, Germany	0	0	**0.000**
Syringic acid	198.2	Sigma, USA	14	45	**0.311**
Tannic acid	1791.2	Sigma, USA	30	127	**0.236**
(+)-Taxifolin	304.3	Extrasynthese, France	20	500	**0.040**
Thymol	150.2	Riedel-de Haën, Germany	13	445	**0.029**
3,4,5-Trimethoxybenzoic acid	212.2	Sigma, USA	1	187	**0.005**
6-Hydroxy-2,5,7,8-tetramethylchroman-2-carboxylic acid	250.3	Aldrich, Germany	N/A	N/A	
Umbelliferone	162.1	Sigma, USA	22	417	**0.053**
Vanillic acid	168.2	Fluka, Switzerland	6	608	**0.010**
Vanillin	152.2	Merck, Germany	13	331	**0.039**
Butylated hydroxytoluene	220.4	Sigma, Germany	37	861	**0.043**
4-Methylumbelliferone	175.1	Extrasynthese, France	18	590	**0.031**
Isoscopoletin	192.2	Extrasynthese, France	2	41	**0.049**
Fraxetin	208.2	Extrasynthese, France	15	276	**0.054**
Fraxidin	222.2	Extrasynthese, France	0	0	**0.000**
Daphnetin	178.4	Extrasynthese, France	27	240	**0.113**
Daphnetin-7-methylether	192.2	Extrasynthese, France	0	0	**0.000**
Scopoletin	192.2	Sigma, USA	33	639	**0.052**
6-Methylcoumarin	160.7	Extrasynthese, France	0	302	**0.000**
4-Hydroxycoumarin	162.1	Extrasynthese, France	4	110	**0.036**
Warfarin	308.3	Sigma, USA	0	378	**0.000**
6-Methoxy-4-methylcoumarin	190.2	Extrasynthese, France	6	739	**0.008**
7-Methoxy-4-methylcoumarin	190.2	Extrasynthese, France	3	242	**0.012**
5,7-Dihydroxy-4-methylcoumarin	193.2	Extrasynthese, France	6	323	**0.019**
6,7-Dihydroxy-4-methylcoumarin	193.2	Extrasynthese, France	4	75	**0.053**
Herniarin	176.2	Extrasynthese, France	18	618	**0.029**
6,8-Dibromocoumarin carboxylic acid	348	Avocado, UK	N/A	N/A	
Esculetin	178.2	Fluka, Switzerland	32	755	**0.042**
Esculin sesquihydrate	367.3	Fluka, Switzerland	1	163	**0.006**

Table S1. *Cont.*

**Chemical name**	**MW**	**Supplier**	**active**	**total**	**ratio (actuve/total)**
Citropten	206.2	Extrasynthese, France	3	368	**0.008**
Xanthotoxin	216.3	Extrasynthese, France	117	1146	**0.102**
Coumarin 106	281.4	Acros, USA	2	2	**1.000**
Coumarin 153	302.2	Acros, USA	79	619	**0.128**

"Supplier" indicates the provider of the compound used in the current study. Bioactivity summary data were collected from PubChem database in July 2012. "N/A" indicates that the compound was not found in PubChem BioAssays database.
